# Rapeseed oil fortified with micronutrients can reduce glucose intolerance during a high fat challenge in rats

**DOI:** 10.1186/s12986-018-0259-x

**Published:** 2018-03-20

**Authors:** Frederic Capel, Alain Geloen, Carole Vaysse, Gaelle Pineau, Luc Demaison, Jean-Michel Chardigny, Marie-Caroline Michalski, Corinne Malpuech-Brugère

**Affiliations:** 1 INRA, UNH, Unité de Nutrition Humaine, CRNH Auvergne, Université Clermont Auvergne, 58 rue Montalembert - BP 321, F-63000 Clermont-Ferrand, France; 2 Laboratoire CarMeN, INRA UMR1397, INSERM U1060, Univ-Lyon, Université Claude Bernard Lyon 1, INSA-Lyon, IMBL, F-69621 Villeurbanne, France; 3 ITERG-ENMS, Université de Bordeaux, rue Léo Saignat, 33076 Bordeaux Cedex, France; 4 Present address : Centre de Recherche INRA Bourgogne Franche Comté Bâtiment Le Magnen, 17 rue Sully BP 86510, 21065 DIJON Cedex, France

**Keywords:** Fatty acids, Rapeseed oil, Micronutrients, Metabolic syndrome, Obesity

## Abstract

**Background:**

Better choices of dietary lipid sources and substitution of refined by fortified oils could reduce the intake of saturated fatty acids (FA) and increase the intake of omega 3 FA concomitantly to healthy bioactive compounds.

**Methods:**

The development of obesity and metabolic disturbances was explored in rats fed during 11 weeks with a high fat diet (HFD) in which the amount of saturated and polyunsaturated FA was respectively reduced and increased, using rapeseed oil as lipid source. This oil was used in a refined form (R) or fortified (10 fold increase in concentration) with endogenous micronutrients (coenzyme Q10 + tocopherol only (RF) only and also with canolol (RFC)). The effect of substituting palm by rapeseed oil was analysed using a student t test, oil fortification was analysed using ANOVA statistical test.

**Results:**

Despite a similar weight gain, diets R, RF and RFC improved glucose tolerance (+ 10%) of the rats compared to a standard HFD with palm and sunflower oils as lipid source. Plasma glucose was lowered in RF and RFC groups (− 15 and 23% respectively), although triacylglycerol level was only reduced in group RFC (− 33%) compared to R. The fortification with canolol promoted the activation of Akt and AMP-activated protein kinase (AMPK) in skeletal muscle and subcutaneous adipose tissue respectively. Canolol supplementation also led to reduce p38 MAPK activation in skeletal muscle.

**Conclusions:**

This study suggests that the presence of endogenous micronutrients in rapeseed oil promotes cellular adaptations to reverse glucose intolerance and improve the metabolism of insulin sensitive tissues.

**Electronic supplementary material:**

The online version of this article (10.1186/s12986-018-0259-x) contains supplementary material, which is available to authorized users.

## Background

The westernisation of dietary habits in most countries is characterized by an increased intake of energy-dense foods with a high amount of refined sugars, saturated fatty acids (SFA) and an elevated ω6/ω3 ratio. The associated increased prevalence of overweight, obesity and related metabolic disorders and cardiovascular diseases are then a major health challenge. All these events are a leading cause of disability and mortality. Beyond aging or genetic influences, modifiable lifestyle changes could strongly alter the pattern of risk factors, such as plasma lipid and glucose profiles, fat mass accumulation in the visceral area, metabolic flexibility, inflammation and oxidative stress. The consumption of refined oils as lipid source containing high amounts of SFA but lacking bioactive compounds increases the risk of developing of these troubles. The dietary intake of *cis* monounsaturated and polyunsaturated FA, bioactive antioxidants such as vitamins, phenolic compounds is recognized for cardioprotective and healthy metabolic effects [1–3]. These nutrients are mainly found in vegetable oilseeds. Rapeseeds represent a good and sustainable source of ω3 polyunsaturated fatty acids (PUFA) for humans. Oil derived from this plant contains 8–10% of linolenic acid (ALA, 18:3ω3) and has a healthy ω6/ω3 ratio. Its consumption thus contributes to reduce the ω6/ω3 ratio of the diet and to increase the production of long chain PUFA by elongation of ALA into EPA and DHA in biological tissues. It could also contribute to limit the adipogenic and metabolic effects of ω6 PUFAs, such as linoleic acid (LA, 18:2ω6) [4, 5]. Rapeseeds contain bioactive compounds including antioxidant vitamins such as tocopherol (mainly alpha tocopherol), phenolic molecules (canolol, sinapic acid, sinapine), coenzyme Q (CoQ) and phytosterols. These micronutrients have healthy metabolic, anti-inflammatory and physiologic effects [6–10]. The intake of fatty acid (notably PUFA) increases the rate of fatty acid oxidation leading to peroxisomal and mitochondrial hydrogen peroxide production. The intake of antioxydants concomitantly to ω3 PUFA represents a relevant nutritional strategy to reduce obesity-related metabolic disturbances by accommodating changes in antioxidant activities and inflammation [11]. These antioxidants could also facilitate PUFA’s preservation of from beta oxidation and incorporation in biological membranes. Increased cellular level of ω3 PUFA could then protect insulin-responsive tissues from insulin resistance by limiting defects in the cellular signalling pathways activated or repressed by the hormone [12–14]. The presence of micronutrients in the commercialized oil is limited by the technological process of oil extraction and refining [15] but new processes could be used to increase their retention in vegetable oils [16]. The effects of the different bioactive compounds in fortified rapeseed oil (ω3 fatty acids, canolol, CoQ10 and tocopherol) used alone or in combination are only partially characterized in the context of metabolic disorders linked to the excessive intake of SFA. The present study was designed to determine if an increase of the relative amount of ω3 PUFA and bioactive micronutrients could limit the impact of a hypercaloric diet, containing 30% of lipids with a high proportion of SFA on fat mass gain and glucose tolerance.

## Methods

### Oil preparation

Refined palm oil was provided by the société industrielle des oleagineux (Saint-Laurent-Blangy, France), refined rapeseed and safflower oils were prepared by the Institut des Corps Gras (ITERG, Pessac, France). Canolol extract was prepared by a thermal treatment of rapeseed crops by ITERG. Alpha-tocopherol was purchased from Sigma (St. Quentin Fallavier, France) and CoQ10 was a generous gift of Kaneka nutrients (Pasadena, Tx, USA). Fortified rapeseed oil mixtures were prepared to contain 2000 and 300 mg/kg of a-tocopherol and CoQ10 respectively (RF) plus 600 eq. sinapic acid of canolol in RFC oil. The exact compositions are provided in Tables 1, 2, and 3.

**Table 1:** Composition of the high fat diets and detailed composition in micronutrients and FA in the oil mixtures. Composition of the HF diet base

Ingredients (g/100 g)	
Casein	20
L-Cystine	0.3
Sucrose	5
Corn starch	33.7
Pure Cellulose	5
PS, R, RF or RFC oil	30
Mineral mix PM 205B SAFE	5
Vitamin mix PV 200 SAFE	1
*Energy (kcal/kg)*	*5260*
*Lipids (% AET)*	*51*

*Abbreviations: PS* palm/sunflower oil mix, *R* rapeseed oil mix, *RF* rapeseed oil fortified with α-tocopherol and Co-Q mix, *RFC* rapeseed oil fortified with α-tocopherol, Co-Q and canolol mix

**Table 2:** Composition of the high fat diets and detailed composition in micronutrients and FA in the oil mixtures. Micronutrients contents in different HF diets

Ingredients mg/kg	PS	R	RF	RFC
total Tocopherols	53.1	53.5	232.0	268.1
alpha-tocopherol	< 2	< 2	96.2	108.6
Tot tocotrienols	16.8	5.6	56.8	52.8
CoQ9	5.6	1.8	2.5	2.9
CoQ10	29.4	26.6	315.7	308.8
Canolol (eq sinapic ac.)				28.1

*Abbreviations: PS* palm/sunflower oil mix, *R* rapeseed oil mix, *RF* rapeseed oil fortified with α-tocopherol and Co-Q mix, *RFC* rapeseed oil fortified with α-tocopherol, Co-Q and canolol mix

**Table 3:** Composition of the high fat diets and detailed composition in micronutrients and FA in the oil mixtures. Preparation and composition of the different oil mixtures

Components in oil	PS mix	R mix	RF mix	RFC mix
Palm oil	90	60	60	60
Sunflower oil	10			
Rapeseed oil		40	40	40
alpha tocopherol (mg/kg)	125	180	2140	2020
CoQ10 (mg/kg)	20	20	260	260
Canolol (eq. sinapic acid)	–	–	–	600
FA (%)				
C16:0	40	28.3	28.4	28.6
C18:0	4.2	3.2	3.2	3.2
*Sum SFA*	*46.3*	*33.2*	*33.3*	*33.5*
C18:1	43.5	48.7	48.8	48.6
*Sum MUFA*	*43.9*	*49.7*	*49.7*	*49.5*
18:2 n-6	9	13.2	13.1	13.1
18:3 n-3	0.2	3.1	3.1	3.1
Trans FA	0.6	0.8	0.8	0.7

*Abbreviations: PS* palm/sunflower oil mix, *R* rapeseed oil mix, *RF* rapeseed oil fortified with α-tocopherol and Co-Q mix, *RFC* rapeseed oil fortified with α-tocopherol, Co-Q and canolol mix. *FA* fatty acids, *SFA* saturated fatty acids, *MUFA* monounsaturated fatty acids, *Trans FA* trans fatty acids

### Animals and diets

Male Wistar rats of 220–250 g from Janvier SA (Le Genest Saint-Isle, France) were collectively housed (4 per cage) in a temperature controlled room (22 °C) with a 12 h light/12 h dark cycles. After 2 weeks of chow diet, rats were randomly divided into 4 groups fed one of the following HF diets (30% of lipids *w*/w) for 11 weeks: high palm/sunflower oil (PS), high palm/rapeseed (R), high palm/rapeseed oil fortified with alpha tocopherol-CoQ10 (RF) and high palm/rapeseed oil fortified with alpha tocopherol-CoQ10-Canolol (RFC). The composition of the HF diets and the final content in the different compounds are described in Tables 1 and 2. Only the lipid source (oil mixtures as described in Table 3) differed between the 4 groups of animals receiving a high fat diet. A control group of animals was maintained on a standard chow diet (C) to validate the effect of the PS diet on biometric and metabolic parameters. Body weight and food intake were recorded weekly. After 11 weeks of dietary intervention, rats were killed under anaesthesia after an overnight fast, blood was collected in the presence of ethylene-diamine-tetraacetic acid (EDTA) for FA analyses or heparin for antioxidant level assays. Blood samples were centrifuged at 1500 g (10 min, 4 °C) for plasma collection. Plasma; liver, white adipose tissue (WAT) from different depots, muscles, heart and other tissues were collected, weighted and stored at − 80 °C until analyses. All procedures involving animals and their care were approved by the Institutional Animal Care and Research Advisory Committee of the INSA Lyon.

### Glucose tolerance test

After 9 weeks of dietary intervention, animals were fasted for 16 h before an intraperitoneal glucose injection (2 g/kg body weight). Blood glucose was measured at baseline, i.e. 5 min before the injection and 0, 30, 60, 90, 120 and 180 min after. Glucose concentration was measured with a commercial glucometer (Accu-Chek, Roche, France).

### Insulin tolerance test

After 9 weeks of dietary intervention, animals were fasted for 16 h before an intraperitoneal insulin injection (Novo Nordisk, 1 U/kg body weight). Blood glucose was measured at baseline, i.e. 5 min before and 0, 15, 30, 60, 90 and 120 min after insulin injection as described for glucose tolerance test.

### Plasma biochemistry

Plasma levels of glucose, non-esterified fatty acids, glycerol, triacylglycerol and total cholesterol were measured using Konelab TM 20 analyser (Thermo Electron SA, Cergy-Pontoise, France), according to manufacturer’s instruction of each assay. Ferric reducing ability of plasma (FRAP) was evaluated as described previously [17].

### Fatty acids analyses

Total lipids were extracted from tissues and plasma according to Folch et al. [18]. Lipids from red blood cells were extracted according to the method proposed by Peuchant et al. [19]. The organic phase was evaporated under nitrogen and fatty acid methyl esters (FAME) were prepared using sodium methoxide and sulfuric acid [20]. Analytic GC-FID analyses of FAME were performed using a gas chromatograph (Thermo Electron Corporation; Waltham, MA) equipped with a flame ionization detector. Hydrogen was used as carrier gas. FAME were analysed using a BPX 70 capillary column (60 m/0.25 mm internal diameter/0.25 μm film thickness) (SGE; Trajan Scientific Australia Pty Ltd).

### α-tocopherol and CoQ10 quantification

Plasma levels of ubiquinol-10 (UL10), ubiquinone-10 (UN10) and α-tocopherol were determined by high-performance liquid chromatography (HPLC) with coulometric electrochemical detection, according to the modified method of Franke [21]. Briefly, 100 μl of plasma were mixed with a mixture of known concentrations of UL9 and UN9 in refrigerated methanol as internal standards. This mixture was immediately extracted with chilled hexane followed by centrifugation for 5 min at 4 °C and 4000 g, and then analysed by ultra-HPLC (Ultimate 3000 UHPLC system (ThermoFisher) with a EC3000RS Coulochem detector and 6011RS analytical cells set at − 0.70 and + 0.70 V) equipped with a Hypersil Gold C18 column (100 mm × 2.1 mm × 1.9 μm; ThermoFisher), using a mobile phase of (*v*/v) 90% methanol, 6.5% acetonitrile, 1.5% water, and (*w*/*v*) 2% aq ammonium formate at a flow of 0.45 ml/min.

### Western blotting

Tissues were ground three times in a mini bead beater in presence of lysis buffer (50 mM hydroxyethyl piperazineethanesulfonic acid (HEPES), 150 mM sodium chloride, 10 mM EDTA, 10 mM sodium pyrophosphate tetrabasic anhydrous, 25 mM β-glycerophosphate, 100 mM sodium fluoride, 10% glycerol anhydrous) supplemented with phosphatase inhibitors cocktail (Sigma Aldrich). Successive centrifugations were done to collect supernatant. Protein quantification was performed using a BCA protein assay kit (Pierce, Thermo Scientific). Bovine serum albumin (BSA) standard curve and sample preparation and analysis were realized according to manufacturer’s instructions. For protein immunoblotting, 20–30 micrograms of proteins were loaded for separation by SDS-PAGE electrophoresis and transfer on PVDF membranes. Membranes were then immunoblotted with the appropriate antibody to detect glyceraldehyde 3-phosphate dehydrogenase (GAPDH), serine 473 phosphorylated AKT, total AKT. Antibody binding was detected using HRP-conjugated secondary antibodies and ECL western blotting substrate (Thermo Scientific). Immunoblots were visualized by chemiluminescence imaging system (MF ChemiBIS 2020, DNR bio imaging systems, Jerusalem, Israel) and quantified using MultiGauge V3.2 software.

### Gene expression analysis

Total RNA were extracted from tissues using TRIzol® (Thermo Scientific) according to the manufacturer’s instructions. RNA quantification and integrity were verified by measuring the ratio of optical density at 260 nm and 280 nm and by agarose gel respectively. cDNAs were synthesized from 2 μg of total RNA using the High Capacity cDNA Reverse Transcription Kit from Applied Biosystem (Thermo Scientific). The products of reverse transcription were used for Quantitative real time polymerase chain reaction (qRT-PCR) using specific primers and Rotor-Gene SYBR Green PCR master mix on a Rotor-Gene Q system (Qiagen, Courtaboeuf, France). Messenger RNA (mRNA) quantification was assayed using the standard curve of native cDNA and serial dilutions. Primer sequences and PCR conditions are available upon request (frederic.capel@inra.fr). Hypoxanthine guanine phosphoribosyltransferase (HPRT) and Non-POU-domain containing octamer binding protein (NoNo), gene were used as housekeeping gene in skeletal muscle and AT respectively.

### Statistical analyses

All data are presented as means +/− standard error of the mean (SEM) and were analysed using R (Bioconductor). Comparison between control C diet and HF enriched with palm/sunflower (PS) diet was assessed using student *t* test. The effect of refined rapeseed oil in group R was firstly tested in comparison to PS diet using student *t* test. The fortification of rapeseed oil was only analysed by comparing RF and RFC groups to group R using one-way ANOVA and Tukey post-hoc test. In some cases a *t* test was performed between RF or RFC group versus PS group. *P* values below 0.05 were considered significant.

## Results

### Biometric and metabolic changes

Diet PS contained palm and sunflower oils as fat source and thus a high amount of SFA and an elevated LA/ALA ratio of 45 (Table 3). PS and R diets contained 53, and 28.4–35 mg of total tocopherol and CoQ respectively per kg of pellet. RF and RFC diets contained 233–268 and 310 mg of total tocopherol and CoQ respectively per kg of pellet. Canolol was only retrieved in diet RFC which contained 28.1 mg/kg (eq. sinapic acid) of canolol (Table 2). The presence of micronutrients had no significant impact on food consumption (data not shown).

**Fig. 1:**
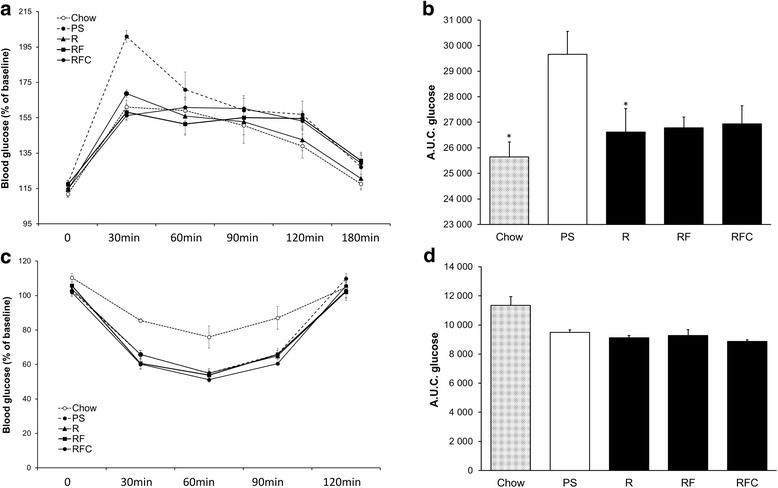
Glucose and Insulin tolerance tests. **a** Glucose tolerance was assessed by measuring blood glucose concentration following intraperitoneal glucose injection to fasted rats after 10 weeks of intervention. **b** Area under the glycaemic curve values from glucose tolerance test. **c** Insulin tolerance was assessed by measuring blood glucose concentration following intraperitoneal insulin injection to fasted rats after 10 weeks of intervention. **d** Area under the glycaemic curve values from insulin tolerance test. Data are means ± SEM of 10 animals per group. (*: *P* < 0.05 vs PS). Abbreviations: *PS* palm/sunflower oil group, *R* rapeseed oil group, *RF* rapeseed oil fortified with a-tocopherol and Co-Q group, *RFC* rapeseed oil fortified with a-tocopherol, Co-Q and canolol group

As reported in Table 4, animals from group PS exhibited significant body weight and fat mass gain compared to group control (C) which was fed with a chow diet. Glucose tolerance was significantly altered (*P* < 0.05, Fig. 1a & 1b) but insulin sensitivity remained unaffected in group PS compared to group C (*P* = ns, Fig. 1c & 1d). Plasma antioxidant capacity estimated by the FRAP was altered in group PS compared to group C (Additional file 1: Figure S1).

**Table 4:** Tissue weights

	Chow		PS		R		RF		RFC		PS vs R	ANOVA
	Mean	SEM	Mean	SEM	Mean	SEM	Mean	SEM	Mean	SEM		R,RF, RFC
Body weight (grams)												
Baseline	285.6	2.14	280.5	5.77	279.5	2.82	278.2	3.54	272.8	3.89	ns	ns
Final	430.8	5.39	474.1^a^	14.17	469.3	13.41	460.2	12.00	461.4	11.07	ns	ns
Organ weights (grams)												
Heart	0.97	0.02	1.00	0.02	0.95	0.03	0.97	0.02	0.99	0.03	*	ns
*Gastrocnemius*	2.37	0.06	2.27	0.05	2.25	0.06	2.22	0.04	2.12	0.06	ns	ns
*Tibialis anterior*	1.49	0.04	1.49	0.04	1.45	0.04	1.43	0.02	1.36	0.04	ns	ns
*Soleus*	0.17	0.01	0.17	0.01	0.17	0.01	0.17	0.01	0.15	0.01	ns	ns
Liver	11.86	0.36	11.02	0.29	11.57	0.45	11.38	0.35	11.74	0.40	ns	ns
Subcutaneous AT	4.07	0.59	8.14^a^	0.79	8.63	0.75	8.44	0.68	8.79	0.71	ns	ns
Retroperitoneal AT	12.72	0.84	22.53^a^	1.45	23.50	1.80	21.00	1.37	22.99	1.42	ns	ns
Epididymal AT	10.01	0.57	17.39^a^	1.38	20.03	1.76	17.16	1.59	18.77	1.25	ns	ns
Mesenteric AT	8.38	0.35	13.21^a^	1.21	13.63	1.16	12.55	1.03	13.13	0.82	ns	ns
Epicardial AT	0.23	0.02	0.31	0.06	0.33	0.04	0.34	0.04	0.32	0.03	ns	ns
Tot fat depots	25.40	1.24	48.38^a^	3.39	46.09	3.58	42.33	2.89	45.23	2.62	ns	ns

Data are mean ± SEM for *n* = 12 per group. a: significantly different from chow (*P* < 0.05); *: *P* < 0.05. Comparison between groups R, RF and RFC were performed by one way ANOVA. *Abbreviations:* *AT*, adipose tissue, Chow standard low fat diet, *PS* palm/sunflower oil group, *R* rapeseed oil group, *RF* rapeseed oil fortified with α-tocopherol and Co-Q group, *RFC* rapeseed oil fortified with α-tocopherol, Co-Q and canolol group

We aimed to evaluate the effects of the substitution of 40% of fat from the PS HF diet by rapeseed oil (diet R). We then obtained a diet exhibiting an enrichment in PUFA and a reduced LA/ALA ratio which as close to 4 (Table 3). Consumption of diet R did not prevented body weight gain (Table 4) compared to diet PS. Furthermore, fat mass, liver, muscles, heart weights were similar between group PS and R (Table 4). Animals receiving diet R were protected from glucose intolerance as compared to group PS (Fig. 1b). Insulin sensitivity was comparable in these two groups (Fig. 1d). After 11 weeks of feeding with each diet, plasma cholesterol, glycerol, triacylglycerol and non-esterified fatty acids (NEFA) concentrations were not different between the two groups (Table 5).

**Table 5:** Plasma parameters and FA composition

	Chow		PS		R		RF		RFC		PS vs R	ANOVA
	Mean	SEM	Mean	SEM	Mean	SEM	Mean	SEM	Mean	SEM		R-RF-RFC
NEFA (mmol/L)	1.56	0.13	0.97	0.11	1.14	0.11	1.38	0.13	1.07	0.14	ns	
TG (mg/dL)	215.49	23.53	188.11	23.97	180.11	15.03	224.81	25.42	130.66	6.84	ns	3
Chol (mg/dL)	87.16	4.59	99.03	5.58	95.37	4.16	86.93	3.84	95.21	3.65	ns	
Glycerol (mmol/L)	0.29	0.04	0.26	0.03	0.34	0.04	0.36	0.03	0.40	0.05	ns	
Glucose (mg/dL)	240.44	5.57	268.95	18.17	298.90	17.57	231.09	3.73	255.23	4.64	ns	1,2
NEFA/glycerol	5.20	0.35	4.09	0.50	3.79	0.53	3.74	0.52	2.56 #	0.24	ns	
FA (%)												
SFA	31.57	2.63	37.16	1.33	31.25	1.99	31.38	0.46	30.09	0.37	*	ns
MUFA	21.15	0.87	33.15	1.48	34.42	1.59	33.06	1.23	30.61	1.01	ns	ns
PUFA (n-6)	43.37	2.35	28.14	1.05	29.45	1.84	30.64	1.32	33.67	1.13	ns	ns
PUFA (n-3)	3.31	0.15	0.57	0.03	4.06	0.15	4.14	0.12	4.33	0.13	*	ns
18:3(n-3)	1.11	0.06	0.09	0.00	1.28	0.12	1.45	0.09	1.25	0.05	*	ns
20:5(n-3)	0.39	0.05	0.04	0.01	0.56	0.06	0.59	0.06	0.62	0.03	*	ns
22:5(n-3)	0.51	0.03	0.10	0.01	0.56	0.06	0.55	0.05	0.62	0.03	*	ns
22:6(n-3)	1.30	0.09	0.34	0.02	1.66	0.11	1.55	0.12	1.84	0.10	*	ns
Trans FA	0.41	0.03	0.47	0.02	0.60	0.05	0.58	0.02	1.07	0.53	*	ns
Micronutrients												
Coenzyme Q10 (nmol/l)	36.84	3.09	68.64	3.17	54.07	4.11	195.31	16.27	377.84	35.10	*	1,2,3
a-tocophérol (μmol/l)	26.39	1.28	8.24	0.45	7.03	0.34	16.38	0.77	13.71	1.03	*	1,2,3

Data are mean ± SEM for *n* = 10 per group. #, *P* = 0.06 vs R; *P < 0.05. 1, significant difference between R vs RF; 2, R vs RFC; 3: RF vs RFC, ANOVA followed by Tukey test. *Abbreviations:* *NEFA* non-esterified fatty acids, *TG* triacylglycerols, *Chol* cholesterol, *FA* fatty acids, *SFA* saturated fatty acids, *MUFA* monounsaturated fatty acids, *PUFA* polyunsaturated fatty acids, *PS* palm/sunflower oil group, *R* rapeseed oil group, *RF* rapeseed oil fortified with α-tocopherol and Co-Q group, *RFC* rapeseed oil fortified with α-tocopherol, Co-Q and canolol group, *Trans FA* trans fatty acids

The effect of the supplementation with bioactive micronutrients was analysed only between groups R, RF and RFC because of the large difference in lipid composition of diets containing rapeseed oil compared to diet PS. Fortification with purified micronutrients in groups RF and RFC induced an increase in plasma of α-tocopherol and CoQ10 content (Table 5) but had no significant effect on plasma FRAP (Additional file 1: Figure S1). Body and organ weights were similar in groups R, RF and RFC (Table 4). Glucose tolerance was similar in R, RF and RFC groups but plasma glucose was lower in RF and RFC groups compared to group R (*P* < 0.05, Table 5). Fortification of rapeseed oil in group RFC decreased plasma triacylglycerol concentration (*P* < 0.05) compared to group R. The NEFA/glycerol ratio did not differ between R and PS and tended to be reduced in animals from group RFC compared to group R. Of note, the independent comparison between RFC and PS groups showed a lower NEFA/glycerol in RFC group (*p* < 0.05).

### FA profiles in tissues

As shown in Tables 5 and 6, plasma and erythrocyte FA profiles reflected the FA profile of the diets. Plasma levels of all ω3 PUFAs were increased and SFA were decreased in group R in comparison to group PS (*P* < 0.05). Total ω3 PUFAs were increased, although ω6 PUFAs were decreased following diet R compared to PS (*P* < 0.05). No differences in total ω3, nor ω6 amounts were found between diets R, RF and RFC in plasma and erythrocytes (Tables 5 and 6). A lower 18:3 ω3 FA was detected in erythrocytes from group RFC compared to group R (Table 6, *P* < 0.05).

**Table 6:** Erythrocyte FA composition

	Chow		PS		R		RF		RFC		PS vs R	ANOVA
	Mean	SEM	Mean	SEM	Mean	SEM	Mean	SEM	Mean	SEM		R-RF-RFC
FA (%)												
SFA	49.26	0.81	48.38	0.46	46.77	1.53	47.59	0.41	48.05	0.40	*	1,2
MUFA	11.71	0.19	16.62	0.40	18.69	0.93	17.09	0.31	16.33	0.25	ns	2
PUFA (n-6)	35.86	0.56	33.42	0.34	29.71	1.84	30.42	0.32	30.60	0.25	*	
PUFA (n-3)	2.80	0.18	1.10	0.03	4.35	0.39	4.44	0.18	4.53	0.09	*	
*18:3(n-3)*	0.15	0.01	0.02	0.00	0.25	0.04	0.22	0.02	0.17	0.02	*	2
*20:5(n-3)*	0.12	0.01	0.03	0.00	0.30	0.03	0.29	0.02	0.31	0.01	*	
*22:5(n-3)*	0.96	0.08	0.35	0.02	1.47	0.13	1.66	0.06	1.69	0.06	*	
*22:6(n-3)*	1.56	0.12	0.70	0.02	2.33	0.24	2.27	0.14	2.35	0.06	*	
Trans FA	0.31	0.02	0.32	0.01	0.34	0.01	0.34	0.01	0.37	0.01	ns	

Data are mean ± SEM of FA relative amount for *n* = 12 per group. *P < 0.05. 1, significant difference between R vs RF; 2, R vs RFC; 3: RF vs RFC, ANOVA followed by Tukey test. *Abbreviations:* *FA* fatty acids, *SFA* saturated fatty acids, *MUFA* monounsaturated fatty acids, *PUFA* polyunsaturated fatty acids, *PS* palm/sunflower oil group, *R* rapeseed oil group, *RF* rapeseed oil fortified with α-tocopherol and Co-Q group; *RFC* rapeseed oil fortified with α-tocopherol, Co-Q and canolol group, *Trans FA* trans fatty acids

ALA and its elongation products, EPA and DHA, were both increased in skeletal muscle lipids in group R compared to PS (Table 7, *P* < 0.05). DHA content was not different in muscle from R, RF and RFC animals, but EPA concentration was lower in group RF compared to group R (*P* < 0.05). Significant accumulation of ALA was also found in adipose tissue from animals receiving diet R compared to diet PS (*P* < 0.05), but the fortification with micronutrients had no effect on ALA incorporation in RF and RFC groups compared to group R (Additional file 2: Table S1).

**Table 7:** FA composition in skeletal muscle lipids

	Chow		PS		R		RF		RFC		PS vs R	ANOVA
	Mean	SEM	Mean	SEM	Mean	SEM	Mean	SEM	Mean	SEM		R-RF-RFC
FA (%)												
SFA	34.77	0.52	38.55	0.58	35.58	1.00	35.57	0.60	33.89	0.87	*	
MUFA	13.73	0.77	21.55	4.08	26.25	3.62	28.32	0.98	30.12	4.25	ns	
PUFA (n-6)	37.27	0.25	30.42	2.48	24.06	1.27	22.95	0.73	23.86	1.57	*P* = 0.06	
PUFA (n-3)	10.68	0.32	6.17	0.85	11.70	1.26	10.91	0.14	10.16	1.52	*	
*18:3(n-3)*	0.45	0.05	0.03	0.02	0.76	0.10	0.79	0.07	0.72	0.12	*	
*20:5(n-3)*	0.08	0.01	0.00	0.00	0.11	0.03	0.00	0.00	0.08	0.02	*	1
*22:5(n-3)*	1.90	0.14	1.08	0.21	2.19	0.28	1.98	0.10	1.90	0.32	*	
*22:6(n-3)*	8.25	0.26	5.07	0.71	8.64	1.07	8.14	0.15	7.46	1.34	*	
Trans FA	0.32	0.09	0.14	0.04	0.08	0.03	0.04	0.04	0.15	0.06	ns	

Data are mean ± SEM of FA relative amount in *gastrocnemius* muscle lipids for *n* = 5 per group. *P < 0.05. 1, significant difference between R vs RF; 2, R vs RFC; 3: RF vs RFC, ANOVA followed by Tukey test. *Abbreviations:* *FA* fatty acids, *SFA* saturated fatty acids, *MUFA* monounsaturated fatty acids, *PUFA* polyunsaturated fatty acids, *PS* palm/sunflower oil group, *R* rapeseed oil group, *RF* rapeseed oil fortified with α-tocopherol and Co-Q group, *RFC* rapeseed oil fortified with α-tocopherol, Co-Q and canolol group, *Trans FA* trans fatty acids

### Insulin signalling and molecular adaptations in skeletal muscle

**Fig. 2:**
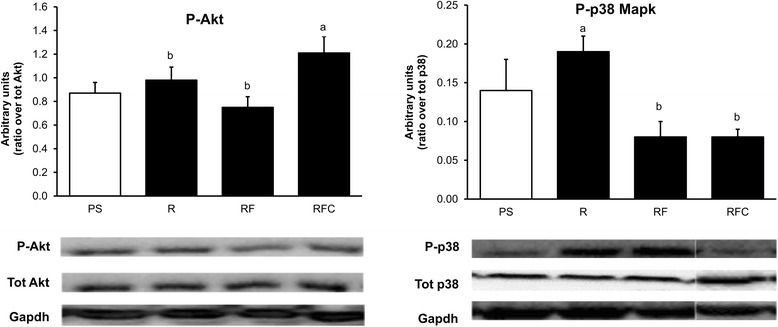
Akt and p38 MAPK phosphorylation in *gastrocnemius* muscle. Data are mean ± SEM for *n* = 8 per group. ANOVA followed by Tukey test was used to compare group R, RF and RFC. Means with the same letter are not significantly different. Abbreviations: *PS* palm/sunflower oil group, *R* rapeseed oil group, *RF* rapeseed oil fortified with a-tocopherol and Co-Q group, *RFC* rapeseed oil fortified with a-tocopherol, Co-Q and canolol group

**Fig. 3:**
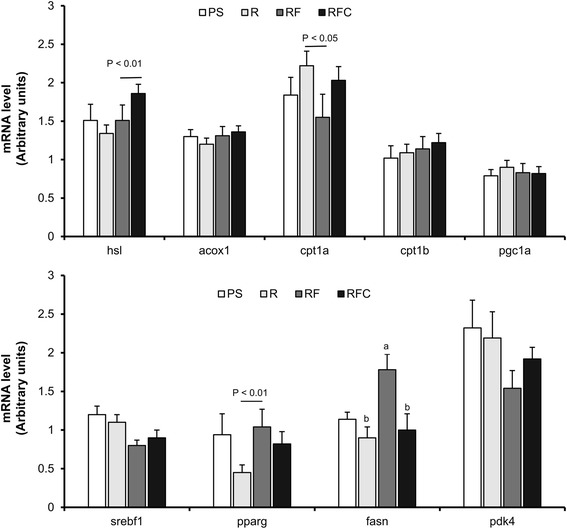
Gene expression in *gastrocnemius* muscle. mRNA levels were normalized to HPRT mRNA level. Data are mean ± SEM for *n* = 10 per group. ANOVA followed by Tukey test was used to compare group R, RF and RFC. Means with the same letter are not significantly different. *Abbreviations:* *PS* palm/sunflower oil group, *R* rapeseed oil group, *RF* rapeseed oil fortified with α-tocopherol and Co-Q group, *RFC* rapeseed oil fortified with α-tocopherol, Co-Q and canolol group

The phosphorylation state of Akt and p38 MAPK in *gastrocnemius* muscle was not different between PS and R groups (Fig. 2). However, Akt phosphorylation was higher in rat's muscle in group RFC compared to group R (Fig. 2, *P* < 0.05). On the contrary, p38 MAPK phosphorylation was decreased in RF and RFC groups compared to group R (Fig. 2, *P* < 0.05). To further explore the molecular adaptations in skeletal muscle, we evaluated the mRNA level of key genes involved in lipid catabolism (acyl-CoA oxidase 1 (ACOX1), carnitine palmitoyl transferase 1a, 1b (CPT1 α & β), hormone-sensitive lipase (HSL), pyruvate dehydrogenase kinase 4 (PDK4), peroxisome proliferator-activated receptor gamma coactivator 1 alpha (PGC1-α)), lipogenesis (sterol regulatory element binding transcription Factor 1 (SREBP1c), proliferator-activated receptor gamma (PPPAR-γ); fatty acid synthase (FASN)) and defense against oxidative stress (catalase, superoxide dismutase 2 (SOD2), glutathione peroxidase 4 (GPX4)) in *gastrocnemius* muscle. Consumption of refined rapeseed oil in group R had no significant effect on the mRNA level of all tested genes compared to group PS (Fig. 3), but some differential regulations were observed in animals receiving fortified oils. FASN and PPPAR-γ mRNA levels were increased in group RF compared to group R (Fig. 3a, *P*< 0.05 and *P* < 0.1 respectively) and the mRNA level of HSL also tended to be elevated in group RFC as compared to group R (*P* < 0.1). Catalase mRNA level was increased in *gastrocnemius* muscle in group RFC compared to group RF and SOD2 mRNA was elevated in skeletal muscle in groups RF and RFC compared to group R (*P* < 0.05).

### Metabolic adaptations in adipose tissue

**Fig. 4:**
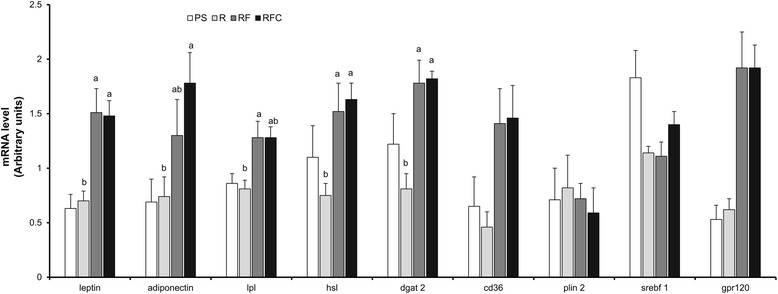
Gene expression in subcutaneous adipose tissue. mRNA levels were normalized to Non-POU domain-containing octamer-binding protein (Nono) mRNA. Data are mean ± SEM for *n* = 10 per group. ANOVA followed by Tukey test was used to compare group R, RF and RFC. Means with the same letter are not significantly different. *Abbreviations:* *PS* palm/sunflower oil group, *R* rapeseed oil group, *RF* rapeseed oil fortified with α-tocopherol and Co-Q group, *RFC* rapeseed oil fortified with α-tocopherol, Co-Q and canolol group

**Fig. 5:**
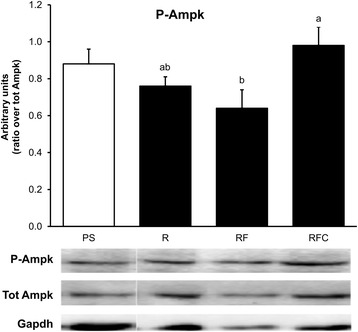
AMPK phosphorylation in subcutaneous adipose tissue. Data are mean ± SEM for *n* = 8 per group. ANOVA followed by Tukey test was used to compare group R, RF and RFC. Means with the same letter are not significantly different. Abbreviations: *PS* palm/sunflower oil group, *R* rapeseed oil group, *RF* rapeseed oil fortified with α-tocopherol and Co-Q group, *RFC* rapeseed oil fortified with α-tocopherol, Co-Q and canolol group

Although no significant changes were reported between R and PS groups, a higher expression level of leptin, adiponectin, G-protein coupled receptor 120 (GPR120), HSL, diacylglycérol O-acyltransférase 2 (DGAT2) and CD36 was observed in subcutaneous adipose tissue from RF and RFC groups in comparison to group R (Fig. 4, *P* < 0.05). No significant modifications were reported in retroperitoneal adipose tissue (data not shown). The activation of AMP-activated protein kinase (AMPK) was evaluated by the quantification of AMPK phosphorylation by western-blot (Fig. 5). An increased phosphorylation level of AMPK was detected in subcutaneous adipose tissue from rat of the RFC group in comparison to group R (Fig. 5).

## Discussion

In the present study, we explored the protective effects of an original enrichment of rapeseed oil with fat soluble micronutrients naturally present in rapeseeds, i.e., α-tocopherol, CoQ10 and canolol against the development of adiposity and its metabolic complications during a HF challenge. An increase of the energy density of the diet, the massive intake of SFA and ω6 PUFAs constitute risk factors for insulin resistance and metabolic disturbances that are linked to fat mass accumulation and inflammation [4, 5]. We used a diet providing approximately 55% of energy from fat with a high proportion of SFA. Even if an increased amount of fat in a normocaloric or hypocaloric diet could have a beneficial effect against obesity and metabolic disorders, hypercaloric, high fat diet is commonly used to promote a rapid increase in fat mass and metabolic abnormalities because of the deleterious effect of SFA and ω6 PUFAs on inflammation, adiposity and metabolism [22–24]. We substituted 40% of palm oil in HF pellets by rapeseed oil with different degrees of fortification. Rapeseed oil contains a high amount of PUFAs and has a healthy ω6/ω3 ratio. We re-introduced purified micronutrients into a refined rapeseed oil in order to calibrate their final concentration, evaluate their respective roles and potential synergistic or complementary health benefits. Both diets contained the same amount of lipids but the percentage of SFA was decreased by 30% in the HF pellets containing rapeseed oil (groups R, RF and RFC), although the amount of MUFA was slightly increased (+ 10%) because of a high amount of oleic acid in rapeseed oil. In the 3 groups of rats fed with a HF diet containing refined (R) or fortified (RF, RFC) rapeseed oil, no difference in weight gain, nor in plasma metabolic parameters were observed compared to a HF diet with high palm oil (PS). However, the substitution of palm by rapeseed oil prevented the development of glucose intolerance after 11 weeks of HFD in rats. This effect was independent of any difference in food consumption which was comparable between groups. Although, tocopherol could induce a transient improvement in glucose homeostasis in overweight subjects receiving a supplementation [25], the fortification with α-tocopherol and CoQ10 with or without canolol had no additional effect on glucose tolerance. Thus, it was likely related to the combined decreased consumption of SFA, the higher intake of ALA and the improvement of the ω6/ω3 ratio. However, the fortification with α-tocopherol, CoQ and canolol (group RFC) had a beneficial effect on triacylglycerol and fasting plasma glucose despite similar changes in the accumulation of ALA elongation/desaturase products (EPA and DHA) compared to groups R and RF in different tissues. The decrease in plasma triacylglycerol level confirms the results obtained in previous studies on the effect of fortified rapeseed oil [6, 7]. Such an effect could be related to changes in the fate of FA in adipose tissue. In agreement with this, the NEFA/glycerol ratio was improved in group RFC. It suggested that adipose tissue from animals receiving the fortified rapeseed oil had a better ability to re-esterify FA, allowing an internal futile cycle [26]. It could be hypothesised that antioxidant intake had facilitated adipose tissue expansion capacity by reducing collagen deposition [27]. Unfortunately, we were not able to evaluate collagen deposition because no tissue was collected for histological analyses but the increase in mRNA levels of several adipogenic genes that we observed support this hypothesis. Antioxidants also induced an increase in leptin and adiponectin expression in adipose tissue which was probably a key element in the effect of ALA intake on the activation of AMPK and its related metabolic improvement [28, 29]. Such cellular adaptations are sensitive to nutritional regulations. Consequently and because our observations were obtained after an overnight fast as generally done in nutritional studies, we cannot rule out that other factors (stress, hormones, locomotor activity) did not interfere with the measurements [30]. It would be thus of interest to confirm these effects after a shorter fasting period (8 h).

In agreement with in vitro observations, the increased expression of PPAR-γ in skeletal muscle that we observed could be related to metabolic improvements [31], such as insulin response as it is well known that ω3 FA are good PPAR-γ agonists. It suggested a stimulation of lipogenesis despite the absence of change in SREBP1c mRNA level. Expression of HSL was also increased in group RFC which lead to suggest a higher lipid mobilization in skeletal muscle. It reinforces the hypothesis of a better FA turnover and probably re-esterification in animals receiving a fortified oil allowing a better glucose handling and metabolic flexibility. In direct line with these observations, the fortification with the 3 micronutrients decreased p38 MAPK activation and restored insulin signalling as demonstrated by the normalization of Akt protein phosphorylation state. Akt is involved in the anabolic response to insulin and glucose uptake following insulin stimulation. The activation of p38 MAPK was found to mediate the inflammatory response in skeletal muscle leading to muscle atrophy [32] and is activated under lipotoxic stress [14]. Interestingly, the fortification induced an increase in the mRNA levels of antioxidant enzymes, suggesting a protective role in this particular context of an increased FA oxidation when FA availability is high. Beneficial effects of coenzyme Q10 on AMPK and Akt activation were previously observed in other organs or cell types. It was proposed that coenzyme Q10 increases cellular cAMP to stimulate AMPK activity in the liver [33]. Other studies suggested that the activation of Akt protein could be a consequence of an alleviation of oxidative stress by coenzyme Q10 in endothelial cells or in the pancreas [34, 35]. It was also demonstrated that tocopherol could protect the proteins involved in insulin signalling from oxidative damage and thus contribute to a higher Akt activity [36]. Even if the precise mechanisms involved remain unclear, a reduction in oxidative stress, a small improvement in mitochondrial activity could be involved in the observed benefits. It could be, at least partially, mediated by an inhibition of the activity of p38 MAPK, leading to a reduction in cellular stress. The effects of canolol on all these proteins remains unknown, but our results suggested an effect which could be synergistic with the presence of α-tocopherol, coenzyme Q or ALA.

The present study aimed to determine if the deleterious effects of SFA could be, at least partially prevented by an improvement in fat quality, even in the context of an hypercaloric diet. The experimental hypercaloric diet used in the present study contained 30% of fat (55% of daily energy intake) and a low amount of sucrose to compare and analyze the effects of oil quality and oil’s micronutrients. Further studies are now required to verify if these effects in a context that better mimics a typical western diet with a lower amount of lipids and a higher amount of sucrose. It was shown that excessive consumption of sucrose inhibited FA oxidation, increased the accumulation of fat mass and aggravated the effect of the high caloric intake [37].

## Conclusion

In rats receiving a HF diet, substitution of SFA by PUFA and a better ω6/ω3 ratio could prevent glucose intolerance, at least in the short term. Fortification of rapeseed oil with naturally constituting micronutrients could further improve the metabolism of skeletal muscle and adipose tissue, allowing a better handling of FA excess and a lower fasting glycaemia, which could be helpful at the long term. Our results encourage the use of non-refined oils containing ω3 PUFA and micronutrients contained in the seeds.

## Additional files

Additional file 1: **Figure S1.** Ferric Reducing Antioxidant Power (FRAP) in plasma. Data are mean ± SEM for *n* = 12 per group (*P < 0.05 vs Chow). *Abbreviations:* *PS* palm/sunflower oil group, *R* rapeseed oil group, *RF* rapeseed oil fortified with α-tocopherol and Co-Q group, *RFC* rapeseed oil fortified with α-tocopherol, Co-Q and canolol group. (TIFF 241 kb)

Additional file 2: **Table S1.** FA composition in retroperitoneal adipose tissue. (*P < 0.05). 1, significant difference R vs RF; 2, R vs RFC; 3: RF vs RFC, ANOVA followed by Tukey test. Data are mean ± SEM of FA relative amount for *n* = 12 per group. *Abbreviations:* *FA* fatty acids, *SFA* saturated fatty acids, *MUFA* monounsaturated fatty acids, *PUFA* polyunsaturated fatty acids, *Trans FA* trans fatty acids. (DOCX 16 kb)

## Abbreviations

ALA: alpha linolenic acid
AMPK: AMP-activated protein kinase
AT: adipose tissue
C: chow diet
cDNA: complementary desoxyribonucleic acid
DHA: docosahexaenoic acid
EPA: eicosapentaenoic acid
FA: fatty acids
FAME: fatty acid methyl esters
FRAP: ferric reducing ability of plasma
GAPDH: glyceraldehyde 3-phosphate dehydrogenase
HF: High Fat
HPRT: hypoxanthine guanine phosphoribosyltransferase
LA: linoleic acid
NEFA: non-esterified fatty acids
NoNo: Non-POU-domain containing octamer binding protein
PS: high fat diet with palm and sunflower oil
PUFA: polyunsaturated fatty acids
R: high fat diet containing refined rapeseed oil
RF: high fat diet containing refined rapeseed oil fortified with CoQ10 and tocopherol
RFC: high fat diet containing refined rapeseed oil fortified with CoQ10, tocopherol and canolol
RNA: ribonucleic acids
SFA: saturated fatty acids
