# Supplementary figures and images for: Rapeseed oil fortified with micronutrients can reduce glucose intolerance during a high fat challenge in rats
Source: Nutr Metab (Lond). 2018 Mar 20;15:22. doi: 10.1186/s12986-018-0259-x (PMC5859643; doi:10.1186/s12986-018-0259-x)

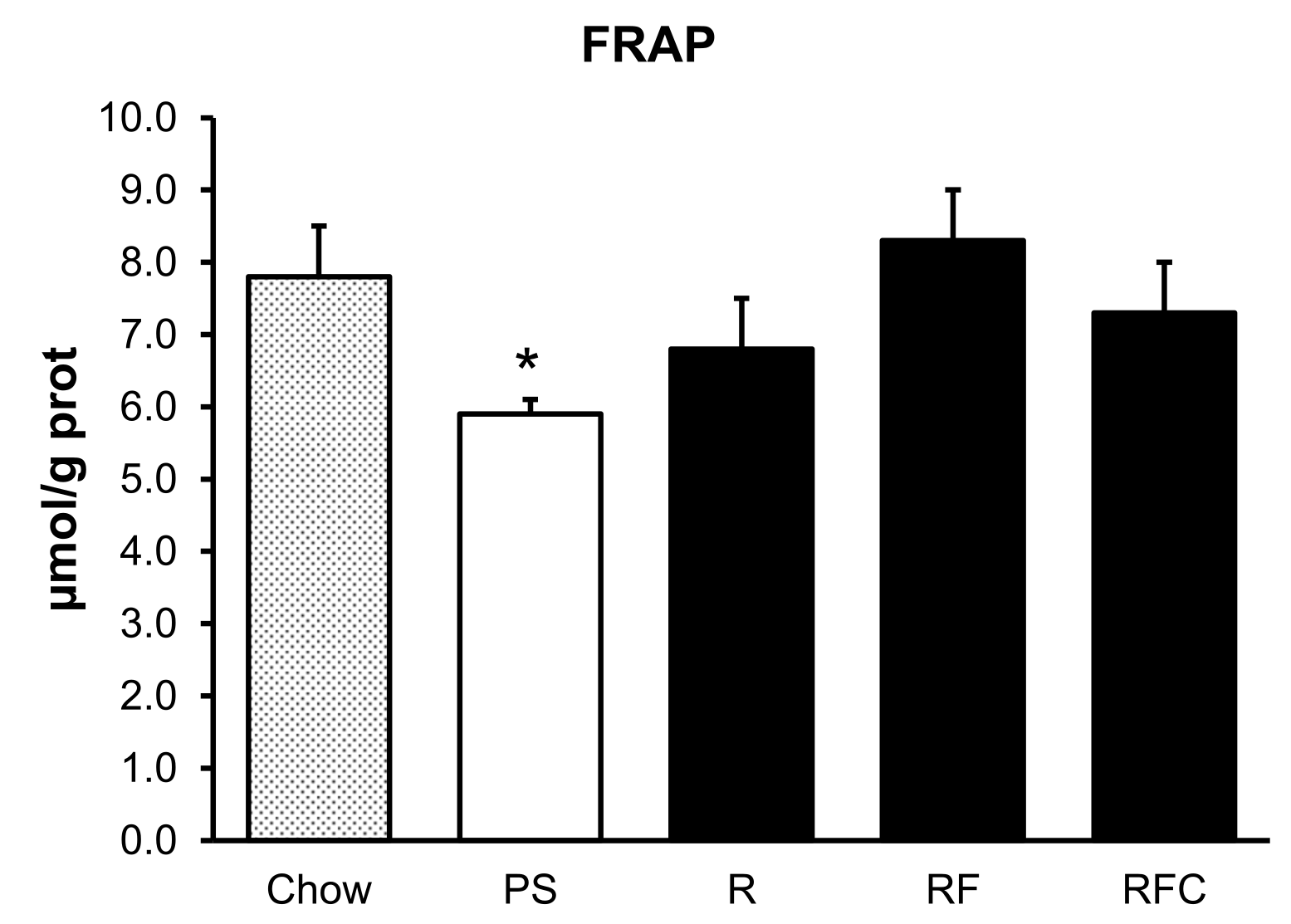

Supplement: Supplementary file 1 — Figure S1. Ferric Reducing Antioxidant Power (FRAP) in plasma. Data are mean ± SEM for n = 12 per group (*P < 0.05 vs Chow). Abbreviations: PS palm/sunflower oil group, R rapeseed oil group, RF rapeseed oil fortified with α-tocopherol and Co-Q group, RFC rapeseed oil fortified with α-tocopherol, Co-Q and canolol group. (TIFF 241 kb) [file 12986_2018_259_MOESM1_ESM.tif]
