# Supplementary material for: Rapeseed oil fortified with micronutrients can reduce glucose intolerance during a high fat challenge in rats
Source: Nutr Metab (Lond). 2018 Mar 20;15:22. doi: 10.1186/s12986-018-0259-x (PMC5859643; doi:10.1186/s12986-018-0259-x)
Supplement: Supplementary file 2 — Table S1. FA composition in retroperitoneal adipose tissue. (*P < 0.05). 1, significant difference R vs RF; 2, R vs RFC; 3: RF vs RFC, ANOVA followed by Tukey test. Data are mean ± SEM of FA relative amount for n = 12 per group. Abbreviations: FA fatty acids, SFA saturated fatty acids, MUFA monounsaturated fatty acids, PUFA polyunsaturated fatty acids, Trans FA trans fatty acids. (DOCX 16 kb) [file 12986_2018_259_MOESM2_ESM.docx]

|  | Chow | | PS | | R | | RF | | RFC | | PS vs R | ANOVA |
| --- | --- | --- | --- | --- | --- | --- | --- | --- | --- | --- | --- | --- |
|  | Mean | SEM | Mean | SEM | Mean | SEM | Mean | SEM | Mean | SEM |  | R-RF-RFC |
| FA (%) |  |  |  |  |  |  |  |  |  |  |  |  |
| SFA | 28.70 | 0.87 | 35.32 | 1.58 | 26.89 | 0.98 | 27.10 | 0.78 | 26.90 | 0.89 | * |  |
| MUFA | 33.28 | 1.18 | 53.93 | 1.97 | 56.14 | 1.08 | 56.07 | 0.82 | 56.39 | 1.05 | * |  |
| PUFA (n-6) | 35.57 | 1.21 | 9.89 | 3.13 | 14.72 | 0.65 | 14.72 | 0.70 | 14.63 | 0.50 | * |  |
| PUFA (n-3) | 1.84 | 0.15 | 0.15 | 0.03 | 1.58 | 0.12 | 1.46 | 0.05 | 1.44 | 0.11 | * | 1,2 |
| *18:3(n-3)* | 1.65 | 0.10 | 0.14 | 0.03 | 1.50 | 0.11 | 1.38 | 0.06 | 1.35 | 0.10 | * | 1,2 |
| *20:5(n-3)* | 0.03 | 0.01 | 0.00 | 0.00 | 0.01 | 0.01 | 0.02 | 0.01 | 0.02 | 0.01 | * |  |
| *22:5(n-3)* | 0.07 | 0.03 | 0.00 | 0.00 | 0.03 | 0.01 | 0.03 | 0.01 | 0.03 | 0.01 | * |  |
| *22:6(n-3)* | 0.09 | 0.03 | 0.00 | 0.01 | 0.03 | 0.01 | 0.03 | 0.01 | 0.03 | 0.01 | * |  |
| Trans FA | 0.44 | 0.07 | 0.50 | 0.03 | 0.47 | 0.04 | 0.45 | 0.04 | 0.46 | 0.02 | ns |  |

**Table S1**: FA composition in retroperitoneal adipose tissue

(*P< 0.05). 1, significant difference R vs RF; 2, R vs RFC; 3: RF vs RFC, ANOVA followed by Tukey test. Data are mean ± SEM of FA relative amount for n= 12 per group. Abbreviations: FA, fatty acids; SFA, saturated fatty acids; MUFA, monounsaturated fatty acids; PUFA, polyunsaturated fatty acids; Trans FA, trans fatty acids.
